# Online Interventions for Family Carers of People with Dementia That Focus on Support Strategies for Daily Living: A Mixed Methods Systematic Review

**DOI:** 10.3390/bs15070863

**Published:** 2025-06-25

**Authors:** Julieta Camino, Ana Paula Trucco, Victoria McArthur, Paul Sugarhood

**Affiliations:** 1 School of Health Sciences, Faculty of Medicine and Health Sciences, University of East Anglia, Norwich Research Park, Norwich NR4 7TJ, UK; 2 Norfolk and Norwich University Hospital, Norwich NR4 7UY, UK; 3 School of Allied and Community Health, London South Bank University, London SE1 0AA, UK

**Keywords:** online intervention, family carers, dementia, upskilling, activities of daily living

## Abstract

This review aimed to identify the available online interventions for family carers of people living with dementia and how effective they are when upskilling carers in how to support activities of daily living. Six databases were searched, and 10 articles with six online programmes were identified. Articles used an RCT design, a mixed approach, and a pre-post test design. Data were synthesised using a convergent integrated approach for mixed-methods reviews. Three overarching themes were identified, focusing on accessibility of the programme, the content and information provided, and the outcomes for the carer and the person with dementia. Online interventions with useful content for family carers of people with dementia are easy to access. However, this did not translate into carers’ outcomes, where mixed results were found. No positive findings were reported for people with dementia in terms of social participation, autonomy or self-management abilities. Development of future online interventions should consider integrating carers’ competence, skills and knowledge alongside daily activities to provide a comprehensive approach when training family carers.

## 1. Introduction

Family carers are the main providers of unpaid support to people living with dementia (Prince & Jackson, 2009; WHO, 2021), which accounts for more than GBP 600 billion every year worldwide (Wimo et al., 2023). The caregiving role includes offering assistance with activities of daily living (ADLs) such as managing finances, shopping, cooking, dressing, bathing and eating (Gaugler et al., 2002). Carers employ different approaches to assistance during these tasks (Camino et al., 2024), and using different strategies was associated with activity performance in people living with dementia in the past (Camino et al., 2021), but carers learn alone (Camino et al., 2024). Their main needs are related to providing care (Holt Clemmensen et al., 2021), for example, being educated to provide support (Bressan et al., 2020) and specifically on ADLs (McCabe et al., 2016). However, family carers often lack the skills and training needed for this role (Georges et al., 2008).

Maintaining participation in ADLs of the person living with dementia is important to delay institutionalisation rates (Luppa et al., 2010; Gaugler et al., 2003) and to improve their quality of life (Chan et al., 2015). Support needed increases as ADL impairment rises, which directly results in increased social support required (Takechi et al., 2019) and associated costs of community care (Jönsson et al., 2006). In addition, deterioration of performance of daily tasks is a predictor of increased carer burden (Germain et al., 2009), also raising the time carers provide supervision and support (Reed et al., 2016).

Home-based non-pharmacological interventions, such as the tailored activity programme (Gitlin et al., 2008) and the community occupational therapy intervention (Graff et al., 2006), that aim to improve activity participation of the person living with dementia, were tested in the past. Positive outcomes were reported, but these programmes are costly and labour-intensive (Bennett et al., 2019), and many carers have no access to this type of support (Brodaty et al., 2005; Lourida et al., 2017; Hodgson & Gitlin, 2021). Online resources have since emerged, meaning that programmes that are based on web or computer applications have been developed to reduce the cost of such interventions and to increase accessibility (Murray et al., 2005) while offering a wider range of support (Finkelstein et al., 2012). Thus, online forums for carers have shown positive results (Leng et al., 2020) and benefits for them (McKechnie et al., 2014), including low cost (Aspvall et al., 2021) and flexible access (Hayden et al., 2012). However, they tend to focus on improving carers’ anxiety and depression (Kishita et al., 2018), reducing behavioural symptoms of the person living with dementia and carers’ burden (Griffiths et al., 2018) and increasing carers’ self-management abilities (Boots et al., 2018; Scheibl et al., 2024) rather than upskilling carers on how to support the person living with dementia with daily activities (Parra-Vidales et al., 2017).

Considering the current costs in dementia care and the growing support carers provide, there is an urgent need to equip them with the skills required to better assist people living with dementia in maintaining ADLs (House of Commons All-Party Parliamentary Group on Dementia, 2011). This aligns with the priorities of people living with dementia of remaining as independent as possible (Kelly et al., 2015) and can potentially reduce institutionalisation due to the daily support people require (Cepoiu-Martin et al., 2016). Online interventions can be a key approach to improving carers’ access to the information and training they need to support their family members with daily activities (Mohr et al., 2018).

This systematic review aims to identify and synthesise the evidence on the effectiveness of the available online interventions for family carers of people living with dementia that target or are designed to provide them with support strategies to assist family members living with dementia with their ADLs.

## 2. Methods

The Preferred Reporting Items for Systematic Reviews and Meta-Analyses (PRISMA) guidelines and recommendations (Page et al., 2021) were followed when designing and conducting this mixed-methods systematic review (PROSPERO Registration number CRD42024562605).

### 2.1. Eligibility Criteria

The Population, Intervention, Comparison, Outcomes and Study (PICOS) framework was used to define the eligibility criteria of this review.

Population: Articles were eligible if they included family carers of people living with any dementia subtype or Mild Cognitive Impairment (MCI). Among these, non-professional volunteers were also eligible if they were part of a family-related sample. When programmes were offered to both the carer and the person living with dementia, the article was included, and the data were analysed separately. When the term carer/s is used, we are referring to family carers of people living with dementia.

Intervention: Studies were included if they presented the development and/or testing of an online, internet-based non-pharmacological intervention to upskill family carers on how best to support the person living with dementia during ADLs. The intervention could be online only, online plus telephone contact, online plus home visit, or online plus telephone contact and home visit.

Comparison: Articles were included with any control condition. Articles with no control group were also included.

Outcome(s): Articles were eligible if they included at least one qualitative or quantitative outcome for the carer that measured their skills, sense of competence or knowledge, and/or the engagement, performance or participation of the person living with dementia in ADLs. For this review, the definition of occupations from the American Occupational Therapy Association (AOTA), Occupational Therapy Practice Framework: Domain and Process Fourth Edition (OTPF-4) (AOTA, 2020) was used. Thus, occupations are those activities that individuals perform daily to occupy time and that bring meaning and purpose to their life (World Federation of Occupational Therapists, 2012). Occupations included in this review were ADLs (personal care tasks and instrumental activities), health management (including self-management abilities), leisure and social participation. Articles that solely focused on the carers’ wellbeing outcomes (e.g., depression, anxiety, burden) were excluded.

Study Design: Articles were included if they were written in English, Spanish or Portuguese and were published in peer-reviewed journals. Study designs included randomised controlled trials (RCTs), quasi-experimental designs, uncontrolled trials, case series or case reports, qualitative articles and mixed-design studies. Dissertations, theses, conference abstracts, book chapters and systematic reviews were not eligible.

### 2.2. Information Sources and Search Strategy

A thorough literature search of articles was conducted in June 2024, and included databases were MEDLINE, CINAHL, ERIC, APA PsycInfo, E-Journals and Scopus. The search strategy included terms that were confirmed with an expert librarian. The complete search terms can be found in the Supplementary Materials (Table S1). Additional hand searches were completed within the included articles.

### 2.3. Selection Process

Retrieved articles were exported and merged using Excel, and duplicates were removed by the first author (JC). Screening by title and abstract for all articles was completed by both the first author (JC) and one co-author (VM). Discrepancies were resolved with one of the other two co-authors (APT, PS) when needed. Full-text screening was completed by two co-authors, and a third was available to assist with disagreements when needed. All co-authors participated in this stage.

### 2.4. Data Extraction

An electronic data sheet was specifically designed and piloted for this review to extract data from the included articles. A meeting was held to discuss disagreements and to finalise the data extraction sheet. Data were then independently extracted by the first author and other co-authors (APT and VM). Discrepancies were discussed, and the opinion from a third author (PS) was sought if needed.

### 2.5. Data Items

The following information was extracted for each individual study: study characteristics (design); participant characteristics (for both carers and the person living with dementia: age, gender, ethnicity, relationship, care duration, dementia subtype and length of symptoms); programme characteristics (aim, description, duration, frequency and setting); and data analysis (quantitative—outcome measures, follow-up period and analysis; qualitative—data collection and analysis methods and findings/themes).

### 2.6. Quality Appraisal

The Joanna Briggs Institute (JBI) Critical Appraisal Tools—Randomised Control Trials (Barker et al., 2023), Qualitative Research (Lockwood et al., 2015), Quasi-Experimental Studies (Barker et al., 2024) and Analytical Cross-Sectional Studies (Moola et al., 2020)—were used to assess the methodological quality of each study. The first author (JC) assessed all included articles with a second assessor (either VM or APT) independently. Disagreements were approached by consensus or with support and the evaluation from a third assessor (PS). Studies were categorised as of high (>70%), moderate (69–50%) and low (<50%) quality according to the Chapter on Assessment of Quality of the JBI Manual for Evidence Synthesis (Aromataris et al., 2024). Studies were not excluded based on their methodological quality, but a report on this assessment is offered in the results section.

### 2.7. Synthesis Methods

The convergent integrated approach for mixed-methods reviews proposed by the JBI (Lizarondo et al., 2020) was used to synthesise the findings of this systematic review. As such, quantitative data were qualitised and thematically merged with the findings from the qualitative analysis. The first author (JC) independently organised and created codes which were then compared and discussed with the second author (APT), using the meta-aggregation approach for qualitative data (Lizarondo et al., 2020). Themes and subthemes were then created and defined in three different meetings between JC and APT. Subsequently, all authors reviewed the themes and subthemes, and names and definitions were finalised. Tables with information about the study, population and interventions’ characteristics were created to summarise the findings and are included in the next section.

## 3. Results

Ten articles testing six different programmes met the eligibility criteria and were finally included in this review. One programme (FindMyApps) was tested in four different articles, and another programme (DEM-DISC) was tested in two articles.

### 3.1. Study Selection

**Figure 1:**
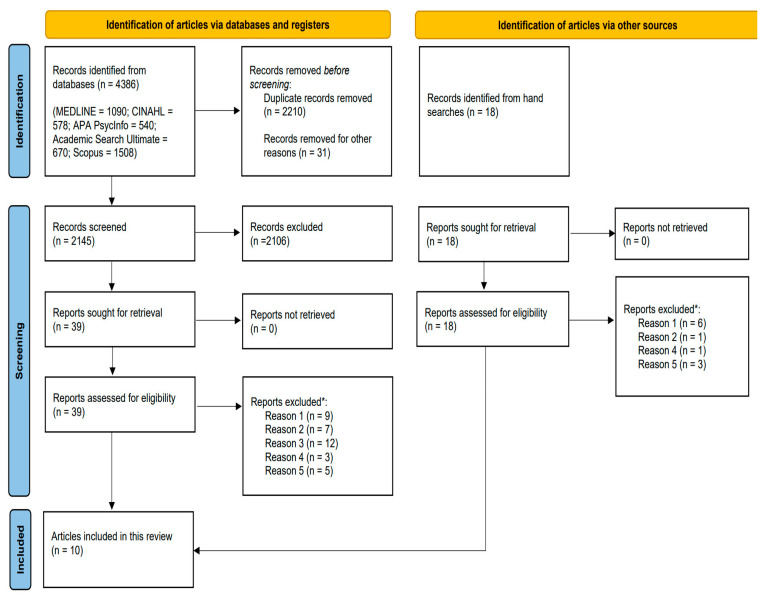
PRISMA 2020 flow diagram for included articles. * Reasons for exclusion: Reason 1: Outcomes do not meet inclusion criteria; Reason 2: Intervention does not meet inclusion criteria; Reason 3: Both outcomes and intervention do not meet inclusion criteria; Reason 4: Population does not meet inclusion criteria; Reason 5: No data reported.

Figure 1 (PRISMA diagram) illustrates the selection process completed in this systematic review. A total of 4386 records were identified initially, of which 2145 were finally screened by titles and abstracts. A total of 57 full-text articles were fully reviewed (18 from hand searches).

### 3.2. Study Characteristics

***Participants***: Table 1 summarises the characteristics of the included articles and participants. Most of the articles (*n* = 8) used an RCT design to test the programme and included either a qualitative or quantitative approach to measure other important elements of the interventions, such as acceptability, feasibility, satisfaction and user-friendliness. The majority of the programmes (*n* = 5) were developed and tested in European countries. One programme was completed in the US. Most interventions were designed to be used by carers. However, one intervention (FindMyApps) was developed to be used by the person living with dementia or MCI with support of their family carer. The majority of the carers were female spouses (71.8%) with an average age of 62 years, although adult children were a majority in two articles (Cristancho-Lacroix et al., 2015; van Mierlo et al., 2015). Most articles included carers looking after people living with different dementia subtypes, including MCI (Neal et al., 2024; Beentjes et al., 2023; Kerkhof et al., 2022; Neal et al., 2023).

***Characteristics of the interventions***: Table 2 includes a detailed summary of the interventions. The majority of the programmes (*n* = 4) used a multicomponent mode of delivery where, in addition to the online content, phone calls by researchers were made or messages in a forum were answered. Programmes included a variety of formats to present the information, including module sections with specific material, videotaped conversations between carers and people living with dementia, areas to post and answer questions, printed manuals and interactive exercises. Outcome measures were predominantly assessed with standardised assessments; however, some articles only focused on non-standardised questions about skills, knowledge and care. Most articles did not report on the frequency that the participants were asked or suggested to use and access the programme. One programme (Neal et al., 2024; Beentjes et al., 2023; Kerkhof et al., 2022; Neal et al., 2023) used a tablet format to train carers and the person living with dementia to find apps that focused on meaningful activities. However, most interventions used an online platform to educate carers on different topics related to dementia with at least one module on practical support or ADLs.

***Control***: All articles but one (Lewis et al., 2010) included a control group. Those articles that reported the use of an app (Neal et al., 2024; Beentjes et al., 2023; Kerkhof et al., 2022; Neal et al., 2023) proposed digital care as usual for the control dyad, where a tablet and training to find links were provided to each control dyad (but without the app installed). One article did not mention the type of usual care their control group received even though they recruited participants from two different countries (Hattink et al., 2015). Another article reported that the control group maintained their usual lifestyle, but information about care received was not given (Núñez-Naveira et al., 2016). The other three articles (Cristancho-Lacroix et al., 2015; van der Roest et al., 2010; van Mierlo et al., 2015) explained that control participants received usual care or had access to traditional channels of information.

***Outcome:*** Carers’ competence was the most common measure included in the programmes. One article included non-standardised questions about carers’ skills and knowledge (Lewis et al., 2010), while others used a non-standardised measure of knowledge about dementia (Cristancho-Lacroix et al., 2015) or about care and services (van der Roest et al., 2010). One programme (Neal et al., 2024; Beentjes et al., 2023; Kerkhof et al., 2022; Neal et al., 2023) included a variety of outcome measures that focused on both the carer’s competence and the social participation, health state (that included ADLs), experienced autonomy and self-management abilities of the person living with dementia. These were all standardised assessments.

### 3.3. Risk of Bias in Studies and Quality of the Evidence

The methodological quality assessment for all articles can be found in the Supplementary Material (Tables S2–S5). Tables are presented according to the study design. The quality of the articles included in this review varied from moderate to high quality. All the studies that used a randomised design explained their randomisation approach, but none of their participants were blinded to the intervention, due to the nature of the programmes. For some articles, important information about assessors (Cristancho-Lacroix et al., 2015; Hattink et al., 2015; van Mierlo et al., 2015) and those delivering the intervention (all articles) as well as treatment of the groups (Cristancho-Lacroix et al., 2015; Hattink et al., 2015) was either unclear or not reported. For those articles using a qualitative approach, there was congruency between the research methodology and the research questions. However, the researcher’s influence on the findings was not always clearly stated (Cristancho-Lacroix et al., 2015; Lewis et al., 2010; Kerkhof et al., 2022).

## 4. Results of Syntheses

**Figure 2:**
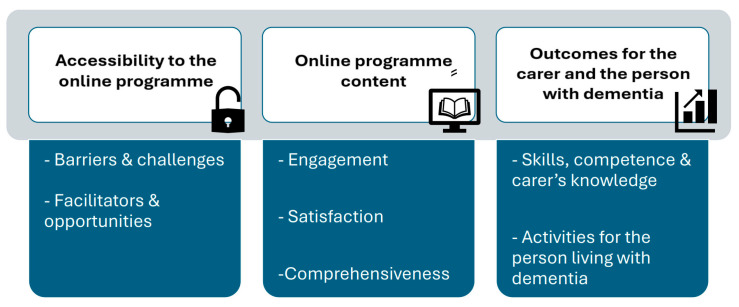
Themes and subthemes.

Three overarching themes were generated to describe the available online interventions for family carers of persons living with dementia that focus on support strategies for ADLs. Figure 2 illustrates the themes and subthemes, and descriptive quotes can be found in Table S6 in the Supplementary Material. The three distinctive themes were (1) accessibility to the online programme; (2) online programme content and (3) outcome for the carer and the person with dementia.

### 4.1. Accessibility to the Online Programme

This theme describes carers’ experiences and perceptions when accessing and using the programme, including both positive and negative aspects of it. The theme comprises two subthemes, namely Barriers and challenges and Facilitators and opportunities.

*Barriers and challenges.* Carers expressed negative experiences and perceived challenges when using the programme. These included technical difficulties, layout, type of design and format interactions and specific ways to access the programme. Carers experienced technical difficulties (Lewis et al., 2010), while some specifications were found inappropriate (Núñez-Naveira et al., 2016). Other carers found it difficult to learn how to use the programme (Kerkhof et al., 2022). Lack of interaction with others (Lewis et al., 2010) and lack of time to use the programmes (Kerkhof et al., 2022) were seen as a problem.

*Facilitators and opportunities.* This subtheme described favourable aspects that enabled and enhanced carers’ positive experiences, including flexibility and convenience of accessing the programme, clarity of instructions and user-friendliness. Many directions were clear (Lewis et al., 2010), and carers found the programmes easy to learn (Kerkhof et al., 2022; van Mierlo et al., 2015) and user-friendly (van der Roest et al., 2010; van Mierlo et al., 2015). The possibility of accessing at their own convenience was seen as a positive aspect of the programme (Lewis et al., 2010). People living with dementia found the programme easy to remember (Kerkhof et al., 2022), and carers’ interest in this type of programme increased after the intervention (Kerkhof et al., 2022).

### 4.2. Online Programme Content

This theme encompasses family carers’ experiences with the content of the programme. It explores how carers received the content and how it met their expectations and contributed to their sense of achievement. This theme comprised three subthemes that described the participants’ connection with the content of the programme, including Engagement, Satisfaction and Comprehensiveness.

*Engagement*. This subtheme pictured the extent to which carers actively interacted with and revisited various sections of the platform, indicating content relevance, interests, motivation and commitment. Carers engaged with the content (Cristancho-Lacroix et al., 2015; van der Roest et al., 2010), and this was relevant to them (Hattink et al., 2015).

*Satisfaction*. Carers’ level of contentment and perceived usefulness of the platform, including positive and negative appraisals on the programme’s structure and content, were important elements of this subtheme. Carers reported being not satisfied with the programme (Núñez-Naveira et al., 2016) or neutral (van der Roest et al., 2010), while others were satisfied with the intervention (van der Roest et al., 2010; Kerkhof et al., 2022; van Mierlo et al., 2015). Level of satisfaction was also seen when carers said they would recommend the programme to a friend (Kerkhof et al., 2022).

*Comprehensiveness*. This relates to the extent to which the platform provided comprehensive and valuable information that met carers’ needs, including the novelty of the content, suitability and amount of information provided. Carers found the strategies given very useful (Lewis et al., 2010), and information about practical difficulties was welcomed by them (Hattink et al., 2015).

### 4.3. Outcome for the Carer and the Person with Dementia

This theme explores and summarises the various outcomes for both carers and the people living with dementia as a result of completing the programme. This theme was characterised by two subthemes, namely Skills, competence and carer’s knowledge and Activities for the person living with dementia.

*Skills, competence and carer’s knowledge*. This subtheme included outcomes for carers, including gaining skills, competence and knowledge. Competence was a recurrent outcome in most programmes. The programme in Lewis et al. (2010) was effective in providing carers with new ideas on how to care for someone living with dementia, and they also gained new skills, such as dealing with behaviours associated with dementia problems. However, mixed results were found in relation to the carer’s competence. For example, carers’ knowledge improved after the intervention (Neal et al., 2024; van der Roest et al., 2010; Neal et al., 2023; van Mierlo et al., 2015), while it did not increase in many others (Cristancho-Lacroix et al., 2015; Hattink et al., 2015; van der Roest et al., 2010; Kerkhof et al., 2022). Knowledge was a less included outcome within the programmes, and it also showed different results. Carers completing some programmes had a better knowledge of dementia (Lewis et al., 2010; Cristancho-Lacroix et al., 2015), but this effect was not sustained after 6 months (Cristancho-Lacroix et al., 2015). Other interventions had no positive effect on carers’ knowledge (van der Roest et al., 2010; Hattink et al., 2015).

*Activities for the person living with dementia*. This subtheme describes the outcomes for the people with dementia in their activities of daily living, including social participation, self-management, health response and autonomy. None of the outcomes included for the person living with dementia improved after the interventions. For example, social participation (Neal et al., 2024; Kerkhof et al., 2022; Neal et al., 2023) and engagement in pleasurable activities (Beentjes et al., 2023; Neal et al., 2023) did not increase after performing the intervention. Health response, including management of ADLs, did not improve (Neal et al., 2024), and the same was seen with the experienced autonomy of the person living with dementia (Kerkhof et al., 2022; Beentjes et al., 2023; Neal et al., 2023). Some articles measured the abilities of the person living with dementia to self-manage, and although there was no improvement in this area (Beentjes et al., 2023; Neal et al., 2023), effect sizes indicated a potential positive influence of the programme on the person’s ability to self-manage ADLs (Kerkhof et al., 2022).

## 5. Discussion

This review aimed to synthesise the current evidence on the available online, non-pharmacological interventions for family carers of people living with dementia that focus on support strategies for daily living. Database searches identified 10 articles in which six programmes were developed and evaluated using different study designs. Six articles used an RCT design, three articles used a mixed approach, and one used a pre-post test design. Methodological quality varied, with most articles presenting a moderate value, where the majority of RCTs did not report on assessors and researchers’ involvement and awareness of the intervention.

A variety of programmes and different ways of delivering online interventions for carers were identified, including web platforms and applications that could be used in a desktop computer or laptop, a tablet and/or a smartphone. Carers found online interventions easy to use, accessible and convenient in terms of accessing them. This supports previous studies on the potential benefits of using online systems to provide carers with support and information they need (Hayden et al., 2012). Yet, positive outcomes identified in this review were mostly seen in those programmes where a multicomponent mode of delivery (for example, online plus phone calls) was used, suggesting that carers still appreciate and benefit from professional support and interaction with others. Downsides of using a web platform with family carers were also identified, including unacceptable format, technical problems and difficulties in learning how to use the systems. This warrants participants’ involvement in the future design and development of this type of programme (Meiland et al., 2017).

Effectiveness outcomes included in this review were selected as eligibility criteria to reflect on the potential programme’s ability to upskill carers on support strategies for daily living. Thus, programmes that included competence, skills and knowledge as carers’ outcomes were eligible, as those which included any outcome on ADL participation or performance of the person living with dementia. Competence, skills and knowledge are interlinked concepts and reflect on personal and technical abilities needed to carry out tasks and perform roles (Schwarz & Wojtczak, 2002). A notable finding of this review is that none of the included articles employed these interrelated outcomes to assess the effectiveness of their interventions in training carers on how to support ADLs. This indicates a significant gap in the current body of research, where traditional outcome measures focus solely on one component, either ADL performance of the person living with dementia or the carer’s competence when supporting their family member. This fragmented approach limits our understanding of the relationship between carer support and meaningful outcomes for the person with dementia, underscoring the need for validated, integrated measurement tools. The absence of a comprehensive approach when developing non-pharmacological online interventions for carers of people living with dementia suggests that existing programmes may not fully address the multidimensional nature of caregiving.

Further, carers’ skills, defined as a special ability to do something after some practice (Cambridge Advanced Learner’s Dictionary & Thesaurus, 2020), were the least reported outcome across the included programmes. Information about skills was broad, and non-standardised questionnaires were used and were related to specific dementia symptoms rather than technical or personal abilities to help with daily tasks. This highlights the research gap between skills needed to provide effective care and the carers’ role in providing support with ADLs.

In contrast, carers’ knowledge was more commonly used as a primary outcome for some articles. Results were inconsistent, showing no improvement in most programmes or decreased knowledge after six-month follow-up. One possible explanation is that knowledge about dementia or care need can vary considerably depending on the stage and severity of the dementia. However, severity of dementia or length of symptoms were not consistently reported in the included articles. Nevertheless, an updated programme (van Mierlo et al., 2015) gave the option of including the severity of the cognitive impairment of the person living with dementia to offer tailored strategies and tips. Nonetheless, information about the participants’ condition, such as dementia stage, was not included in the study (van Mierlo et al., 2015), resulting in a lack of valuable data about offering personalised support to carers using online interventions.

Carers’ sense of competence can be understood from multiple perspectives. It can refer to the carers’ ability to assess their own caregiving performance (Skaff & Pearlin, 1992) or to their perceived confidence in managing the demands of their role (Vernooij-Dassen et al., 1996). Thus, questionnaires developed to measure carers’ sense of competence rely heavily on items related to the burden associated with being a carer (Vernooij-Dassen et al., 1999) rather than on how competent carers are when supporting people living with dementia with daily tasks. This was confirmed in this review, where the results related to sense of competence were contradictory across all the interventions. Although carers identified support strategies for practical difficulties the most useful modules, their sense of competence still did not improve after the intervention (Hattink et al., 2015; Kerkhof et al., 2022; Beentjes et al., 2023). This highlights the need to concentrate efforts on upskilling carers to use support strategies for ADLs, while using outcome measures that can reflect a comprehensive approach to competence and skill development for family carers.

This review found that social participation, self-management and engagement in pleasurable activities were the most reported ADL outcomes for people living with dementia. Notably, the only programme (tested in four different articles) that included outcomes for people living with dementia was the one that tailored the intervention to both the person living with dementia and their family carer (Neal et al., 2024; Beentjes et al., 2023; Kerkhof et al., 2022; Neal et al., 2023). No improvements were found in any of the ADL outcomes. However, one study estimated the effect sizes indicating a possible positive influence of the intervention on self-management and engagement in meaningful activities (Kerkhof et al., 2022). This is not surprising, as maintaining or sustaining participation in different ADLs has been shown to be challenging when working with people living with dementia (Farias et al., 2003; Graff et al., 2006). One possible explanation relies on the progressive nature of dementia, where need for assistance increases over time (Cummings et al., 2023). Online interventions to upskill family carers may need to involve behaviour change techniques (Mohr et al., 2014) so that carers not only improve their knowledge but can use new skills to increase their competence when supporting ADLs. This should also take into consideration cognitive changes in people living with dementia and their care needs.

The content of the majority of the identified programmes included modules with information and videos, while others focused on facilitating navigation of the health and social care system or improving access to meaningful activities. Carers expressed mixed opinions and contradictory views about the value and relevance of the information provided when completing the interventions. Interestingly, although some carers found content appropriate and useful, this was not reflected in the outcome measures used in some of the programmes. This reinforces the idea that providing information for carers does not guarantee improvements in knowledge or acquisition of specific caring skills. This also aligns with the premise that carers need support to help people living with dementia remain living at home (Dawson et al., 2015) in the case where upskilling carers to support daily tasks takes time and carers progressively learn from their own personal experiences (Camino et al., 2024). Nevertheless, carers play a fundamental role when implementing strategies to deal with issues related to ADLs (Camino et al., 2021), which suggests that they can be trained effectively to provide tailored support to their family member. Thus, future research should promote the development and testing of online interventions specifically designed to upskill carers on support strategies for daily activities.

There was considerable heterogeneity in the interventions and study designs, and this was also observed in the variety of results obtained among the carer outcomes. This can be explained by the diverse and dynamic nature of the role of the family carer when supporting people living with dementia in daily tasks. The types of daily activities affected by dementia are diverse and can change over time, requiring that carers adapt continually. In response, different approaches (Vikström et al., 2005; Camino et al., 2024) and strategies (Camino et al., 2021; Burridge et al., 2024) can be employed by carers during ADLs. Consequently, expecting a single outcome measure or a single training approach to capture the complexity of caring in dementia may be unrealistic. Instead, interventions should consider flexibility and personalisation, recognising that carers’ needs and experiences vary significantly and change over time.

It is important to mention some limitations of this systematic review, such as the inclusion of the outcome measures used in the interventions under review. There may be other online interventions with practical modules to upskill carers of people living with dementia that may have considered other outcomes, such as self-efficacy and coping skills. Self-efficacy refers to the carer’s beliefs that they can respond and perform confidently to a specific situation (Bandura, 1977), while coping (Pearlin & Schooler, 1978) refers to those skills carers use to avoid being affected by their situation. Although these may seem relevant skills carers can use when dealing with ADLs, they have been associated with carers’ well-being and may indicate a psychological ability rather than a practical skill. Thus, articles which mainly used coping and self-efficacy were excluded in this review. Future research could consider integrating all relevant domains, such as self-efficacy and coping skills, while also targeting skill development, competence and knowledge to support ADLs, thereby developing holistic models that address the multifaceted needs of carers and people living with dementia.

The inclusion of articles written only in English, Spanish and Portuguese may have excluded other programmes developed in other countries. Further research is needed to understand what other countries are doing to upskill family carers of people living with dementia on support strategies for ADLs.

Methodological inconsistencies, such as the lack of reporting on assessor blinding in RCTs, may impact the reliability of findings. Additionally, the limited inclusion of persons with dementia and their demographic characteristics, including their ethnicity, alongside their carers suggests a gap in dyadic interventions. New online programmes using advanced technologies could incorporate algorithms to tailor responses so that the background and personal circumstances of carers and people living with dementia can be taken into consideration when learning new skills.

## 6. Conclusions

Available online interventions for family carers of people living with dementia do not focus on training carers to provide support on ADLs. Future intervention development should include the carers’ perspectives and views when designing online programmes. This could also consider the carers’ needs when facing the complexities of ADL changes due to the progressive nature of the disease. Finally, future research should also contemplate integrating outcome measures that capture carers’ competence, skills, and knowledge, alongside the ADL performance of people living with dementia, to ensure a more complete understanding of intervention effectiveness and to promote a truly person-centred, dyadic approach to care.

## Figures and Tables

**Table 1:** Characteristics of included articles and participants (n = 10).

Programme, Author, Year & Country	Study Design	Carer Sample (Started/Finished)	PwD Sample	Carers’ Age (SD)	Carers’ Gender (Female %)	Relationship	PwD’s Age (SD)	PwD’s Gender (Female %)	Dementia Subtype (%)
Diapason Cristancho-Lacroix et al. (2015) France	Pilot RCT + acceptability	I: 25/17 C: 24/20	N/A	I: 64.2 (10.3) C: 59.0 (12.4)	I: 64% C: 67%	I: Spouses: 36%; Children: 64% C: Spouses: 46%; Children: 54%	N/A	N/A	AD: 100%
Internet-Based Savvy Caregiver Lewis et al. (2010) USA	Development + Feasibility	I: 47 No control group	N/A	I: 55 (9)	I: 85%	N/R	N/A	N/A	N/A
UnderstAID Núñez-Naveira et al. (2016) Spain, Poland & Denmark	Pilot RCT	I: 36/30 C: 41/31	N/A	Range 25–88 years No mean reported I or C: N/R	I: 70% C: 58.1%	All: no consanguinity (47.8%); with consanguinity (52.2%) I or C: N/R	N/A	N/A	AD: 53%; Other: N/R
DEM-DISC van der Roest et al. (2010) The Netherlands	Pilot pretest–posttest control group	I:14 C:14	I: 12/9 C: 11/11	I:60.2 (14.3) C: 69.9 (13.2)	I: 64.3% C: 92.9%	I: Spouse: 14.3%; Child: 64.3%; Others: 21.4% C: Spouse: 64.3%; Child: 21.4%; Others: 14.3%	I: 83.3 (6.2) C: 80.6 (4.4)	I: 21.4% C: 71.4%	I: AD: 50% VD: 7.2%; MD: 21.4%; Other: 21.4% C: AD: 57.1%; VD:14.3%; MD: 21.4%; Other: 7.2%
DEM-DISC van Mierlo et al. (2015) The Netherlands	RCT	I: 41/38/30 (dyads) C: 32/26/19 (dyads) *	N/A	I: 63.0 (11.6) C: 60.4 (12.7)	I: 61.0% C: 50.0%	I: Spouse: 36.6%; Child: 53.7% C: Spouse: 31.2%; Child: 56.2%	I: 82.1 (7.3) C: 79.5 (7.9)	I: 78% C: 65.6%	I: AD: 43.9%; VD: 12.2%; MD: 14.6% Other: 29.3% C: AD: 43.8%; VD: 15.6%; MD: 15.6%; Other: 25%
STAR E-Learning Hattink et al. (2015) The Netherlands & UK	RCT	I: 27 (laypeople, includes volunteers and family carers) C: 32 (laypeople)	N/A	I: 52.93 (11.43) C: 54.69 (14.36)	I: 74% C: 69%	I: Partner: 33%; Child 30%; Other: 15%; NA 22% (volunteers) C: Partner 28%; Child 16%; Other 33%; NA 22%	N/A	N/A	N/A
FindMyApps Kerkhof et al. (2022) The Netherlands	Exploratory Feasibility RCT	I: 10/7 C: 10/7	I: 7 C: 4	I: 63.0 (11.8) C: 61.0 (11.7)	I: 100% C: 86%	I: Spouse: 86%; Child: 14% C: Spouse: 57%; Child: 43%	I: 68.9 (14.0) C: 76.0 (4.2)	I: 86% C: 50%	I: AD: 43%; VD: 29%; Other: 28% C: AD: 25%; Other: 75%
FindMyApps Beentjes et al. (2023) The Netherlands	Exploratory Pilot RCT	I: 28/25 C: 30/22	I:27/25 C:28/22	I: 65.61 (10.19) C: 68.03 (11.67)	I: 71.4% C: 58%	I: Partner: 82.1%; Child: 14.2%; Other: 3.6% C: Partner: 87%; Child: 13%	I: 72.71 (7.78) C: 71.74 (9.64)	I: 57% C: 64.5%	I: 2. AD 61% 3. VD 11% 5. Other 28% C: AD: 51.6%; VD: 12.9%: Other: 35.5%
FindMyApps Neal et al. (2023) The Netherlands	Superiority RCT	I: 76/64 (dyads) C: 74/64 (dyads) **		I: 64.48 (11.65) C: 61.31 (14.58)	I: 71% C: 76%	I: Partner: 73%; Child: 17%; Other: 10% C: Partner: 72%; Children: 19%; Other: 10%	I: 72.61 (9.51) C: 72.06 (9.22)	I: 55% C: 58%	I: AD: 67%; VD: 19%; Other: 14% C: AD: 47%; VD: 3%: Other: 50%
FindMyApps Neal et al. (2024) The Netherlands	Cost-effectiveness RCT	I: 76 (dyads) C: 74 (dyads) **		I: 65.37 (11.45) C: 62.5 (14.31)	I: 71.05% C: 75.68%	N/R	I: 73.2 (9.48) C: 72.42 (8.77)	I: 44.74% C: 39.19%	Dementia & MCI It does not specify %

* These refers to baseline, 6 months and 12 months follow-up. ** Recruitment of participant dyads took place between January 2020 and July 2022; it does not specify numbers for carers and the person with dementia. Same dataset was used in articles (Neal et al., 2024; Neal et al., 2023), however, Neal et al. (2024) reported demographic information of those participants that completed the programme where Neal et al. (2023) reported demographic information of those who were recruited. AD: Alzheimer’s Disease. VD: Vascular Dementia. MD: Mixed dementia. MCI: Mild Cognitive Impairment. RCT: Randomised Controlled Trial. I: refers to the Intervention group, while C: refers to the control group. N/A: Not applicable. N/R: Not reported.

**Table 2:** Characteristics of the interventions (n = 6).

Programme Name Author, Year & Country	Description and Content	Outcome Measures (Who & Type) (Name & Article When Needed)	Frequency	Mode of Delivery
Diapason Cristancho-Lacroix et al. (2015) France	The Diapason is a fully automated website that includes twelve sessions. Each session provides theoretical and practical information using videos of health professionals, and a practice guide to apply the content to real life situations. Sessions included information about carer stress, understanding the condition and supporting the person with dementia to maintain and improve their autonomy and safety. Other topics included communication, dealing with behaviours, social and financial support, pharmacological and non-pharmacological treatments and the future. Non-mandatory sections included a forum and sections about life experiences and relaxation techniques. One session per week had to be viewed entirely at least once to unblock the next session viewed.	Knowledge about Alzheimer’s Disease (Carer, non-standardised)	Weekly sessions - 15 to 30 mins average & Unlimited access	Online plus online forum to post messages
Internet-Based Savvy Caregiver (IBSC) Lewis et al. (2010) USA	The IBSC was developed as a browser-based computer programme, accessible from any internet-connected computer from a psychoeducational intervention called The Savvy Caregiver Program (Hepburn et al., 2003). Four modules were adapted from this programme: effects of dementia on thinking, taking charge and letting go, providing practical help and managing daily care and difficult behaviour. The programme also provided participants with videotaped conversations between people living with dementia and their carers where they discussed and shared their own stories. Content also included written information about the topics and strategies for carers. A written document with all the content plus descriptions of the videos and instructions about the platform were given to participants.	Caregiving Knowledge & Skills (Carer, non-standardised)	Not reported	Online only
UnderstAID Núñez-Naveira et al. (2016) Spain, Poland and Denmark	The understAID is an application that can be accessed through any device with internet connection such as a smartphone or tablet or through a browser in a personal computer. The application contains different sections (Learning, Daily Task and a Social Network Section) with different information and functions. The Learning area includes five modules with topics including cognitive decline, daily tasks, behavioural changes, social activities and the role of the carer. The content is displayed using text, videos and images and provides links to other websites. The Daily Task section provides a calendar and reminders for appointments and medication. The Social Network section offers a moderated space for carers to interact with other participants to exchange information and suggestions. The understAID offers tailored information using an interactive questionnaire to determine the level of care the person needs, preferences and time available.	Competence (Carers, standardised) CCS	Not reported	Online plus weekly or monthly phone calls (to monitor progress)
STAR E-learning Hattink et al. (2015) The Netherlands and UK	The STAR training portal is a web platform accessible through any internet-based device. It offers an online course with eight modules containing different topics, including information about dementia, diagnosis and daily tasks. It also provides information about communication, behaviours and mood and offers strategies to cope with the condition. The platform uses text, videos and each module offers interactive exercises and knowledge tests in addition to links to other websites.	Knowledge about Alzheimer’s Disease (Carer, standardised) ADKS Competence (Carer, standardised) SSCQ	Own pace	Online only
Dementia-specific Digital Interactive Social Chart (DEM-DISC) * van der Roest et al. (2010) van Mierlo et al. (2015) The Netherlands	DEM-DISC is an internet-based social chart system that provides general and tailored information on available dementia care and welfare services that can be found in specific areas of Amsterdam. DEM-DISC uses a three-step procedure to guide users in understanding their needs. Whenever they had questions about dementia or related needs or care and welfare services, carers in the experimental group could consult DEM-DISC by accessing the system on their own personal computers. The DEM-DISC system provides information on dementia diagnosis, practical support, coping, and finding company. DEM-DISC was adapted (van Mierlo et al., 2015) to be more accessible for the customers. As part of the changes made, users can answer questions about their situation (e.g., severity of dementia, living situation) to offer tailored information about health, care and welfare services for both the person living with dementia and their carers.	Knowledge about care and services (Carer, non-standardised) (van der Roest et al., 2010) Competence (Carer, standardised) SSCQ (both articles)	Not reported	Online plus phone calls for check ups
FindMyApps ** Kerkhof et al. (2022) Beentjes et al. (2023) Neal et al. (2023) Neal et al. (2024) The Netherlands	FindMyApps is a web application where users have access to a database containing pre-selected apps (an app ‘library’). This library contains approximately 180 apps in the domains of self-management and meaningful activities which were assessed as dementia-friendly apps. The apps contain information about self-management and meaningful activities which are considered as dementia-friendly apps. Both the people with dementia and their carers received a 30-min training session on the use of the tablet and the FindMyApps app. Carers were shown how to support their family members using the tablet and the app. Dyads were suggested to practise at least twice a week for the first month. A video with information about the tablet’s functions was shown to participants. This was located in the project’s website for the duration of the intervention. A printed manual was also given to participants with written instructions about the tablet and app and with a list of links to websites with suggested apps for people with dementia or cognitive impairment.	Competence (Carer, SSCQ, standardised) (all articles) Social Participation (PwD, standardised) MSPP (Neal et al., 2024; Beentjes et al., 2023; Neal et al., 2023) ASCOT (Beentjes et al., 2023) Self-Management Abilities (PwD, standardised) SMAS-S (Beentjes et al., 2023) SMAS-30 (Kerkhof et al., 2022) ASCOT (Neal et al., 2023) Participation in daily and social activities (PwD, Standardised) ASCOT (one item) (Kerkhof et al., 2022) PAL (Kerkhof et al., 2022; Neal et al., 2023) Experienced Autonomy (PwD, standardised) EA (Kerkhof et al., 2022; Neal et al., 2023) Health Status-QoL-ADLs (PwD, standardised) EQ-5D-5L (Neal et al., 2024)	On demand	Online plus help desk available, by email or telephone, at any time. Follow-up phone calls with informal carers took place every couple of weeks to provide support if needed.

* Two articles were completed for the same programme. ** Four articles were conducted for the same programme. CCS: Caregiver Competence Scale; ADKS: Alzheimer’s Disease Knowledge Scale; SSCQ: Short Sense of Competence; MSPP: Maastricht Social Participation Profile; ASCOT: Adult Social Care Outcomes Toolkit; SMAS-S: Self-Management Abilities Scale- short version; SMAS-30: Self-Management Abilities Scale-revised 30 items; PAL: Pleasurable Activities List, also called Pleasant Activities List; EA: Experienced Autonomy questionnaire; EQ-5D-5L: EuroQol -Health status, 5 dimensions and 5 levels, quality of life & ADLs.

## Supplementary Materials

The following supporting information can be downloaded at: https://www.mdpi.com/article/10.3390/bs15070863/s1, Table S1: Search Strategy Terms; Table S2: JBI Critical Appraisal Tool for Assessment of Risk of Bias for Randomised Controlled Trials; Table S3: JBI Critical Appraisal Checklist for Qualitative Research; Table S4: JBI Critical Appraisal Tool for Quasi-Experimental Studies; Table S5: JBI Critical Appraisal Tool for Analytical Cross-Sectional Studies; Table S6: Data synthesis: agreed themes sub-themes with definitions and coded items with quotes.
