# Supplementary material for: Online Interventions for Family Carers of People with Dementia That Focus on Support Strategies for Daily Living: A Mixed Methods Systematic Review
Source: Behav Sci (Basel). 2025 Jun 25;15(7):863. doi: 10.3390/bs15070863 (PMC12292812; doi:10.3390/bs15070863)
Supplement: Supplementary file 1 [file behavsci-15-00863-s001.zip › behavsci-3655554-supplementary.pdf]

Supplementary Materials

Table S1. Search Strategy Terms

Database	Search terms	Filter
PsycINFO, CINAHL, MEDLINE, Academic Search Ultimate	Dementia OR Alzheimer* OR “cognitive impairment” OR neurodegenerative OR (memory N2 (loss or problem* or disorder*)) AND Carer* OR caregiver* OR “family member*” OR relatives OR family OR informal AND ADLs OR activit* OR performance OR “daily tasks” OR functioning OR “daily function” OR participation OR tasks OR confidence OR “self-efficacy” OR engagement AND TI (Online OR “web-based” OR internet OR digital OR web) OR AB (Online OR “web-based” OR internet OR digital OR web)	English, Spanish and Portuguese languages
SCOPUS	Dementia OR Alzheimer* OR “cognitive impairment” OR neurodegenerative OR “memory loss” or “memory problem*” or “memory disorder*” AND Carer* OR caregiver* OR “family member*” OR relatives OR family OR informal AND ADLs OR activit* OR performance OR “daily tasks” OR functioning OR “daily function” OR participation OR tasks OR confidence OR “self-efficacy” OR engagement AND Online OR “web-based” OR internet OR digital OR web	

Table S2. JBI Critical Appraisal Tool for Assessment of Risk of Bias for Randomised Controlled Trials

Tool Items	Cristancho-Lacroix, 2015	Neal, 2024	Beentjes, 2023	Núñez-Naveira, 2016	Hattink, 2015	Kerkhof, 2022	Neal, 2023	Van Mierlo, 2015
1. Was true randomization used for assignment of participants to treatment groups?	Yes	Yes	Yes	Yes	Yes	Yes	Yes	Yes
2. Was allocation to treatment groups concealed?	Yes	No	No	Yes	Unclear	Unclear	Yes	Yes
3. Were treatment groups similar at the baseline?	Yes	Yes	Yes	Unclear	Yes	Yes	Yes	Yes
4. Were participants blind to treatment assignment?	No	No	No	No	No	No	No	No
5. Were those delivering the treatment blind to treatment assignment?	No	No	No	No	Unclear	No	No	No
6. Were treatment groups treated identically other than the intervention of interest?	No	Yes	Yes	Yes	Unclear	Yes	Yes	Yes
7. Were outcome assessors blind to treatment assignment?	No	Unclear	Unclear	Yes	Unclear	Yes	Yes	Unclear
8. Were outcomes measured in the same	Yes	Yes	Yes	Yes	Yes	Yes	Yes	Yes

way for treatment groups?								
9. Were outcomes measured in a reliable way	Unclear	Yes	Yes	Yes	Yes	Yes	Yes	Unclear
10. Was follow up complete and if not, were differences between groups in terms of their follow up adequately described and analysed?	Yes	Yes	Yes	Yes	Yes	Yes	Yes	Yes
11. Were participants analysed in the groups to which they were randomized?	Yes	Yes	Yes	Yes	Yes	Yes	Yes	Yes
12. Was appropriate statistical analysis used?	Yes	Yes	Yes	Yes	Yes	Yes	Yes	Yes
13. Was the trial design appropriate and any deviations from the standard RCT design (individual randomization, parallel groups) accounted for in the conduct and analysis of the trial?	Yes	Yes	Yes	Yes	Yes	Yes	Yes	Yes

Table S3. JBI Critical Appraisal Checklist for Qualitative Research

**Tool items**	Cristancho-Lacroix, 2015	Lewis, 2010	Kerkhof, 2022
1. Is there congruity between the stated philosophical perspective and the research methodology?	N/A	N/A	Yes
**2.** Is there congruity between the research methodology and the research question or objectives?	Yes	Yes	Yes
**3.** Is there congruity between the research methodology and the methods used to collect data?	Yes	Yes	Yes
**4.** Is there congruity between the research methodology and the representation and analysis of data?	Yes	Yes	Yes
**5.** Is there congruity between the research methodology and the interpretation of results?	No	Yes	Yes
**6.** Is there a statement locating the researcher culturally or theoretically?	No	No	No
**7.** Is the influence of the researcher on the research, and vice- versa, addressed?	No	No	No
**8.** Are participants, and their voices, adequately represented?	Yes	Yes	Yes
**9.** Is the research ethical according to current criteria or, for recent studies, and is there evidence of ethical approval by an appropriate body?	Yes	Yes	Yes
**10.** Do the conclusions drawn in the research report flow from the analysis, or interpretation, of the data?	Yes	Yes	Yes

Table S4. JBI Critical Appraisal Tool for Quasi-Experimental Studies

Tool Items	van der Roest, 2010
1. Is it clear in the study what is the “cause” and what is the “effect” (i.e. there is no confusion about which variable comes first)?	Yes
2. Was there a control group?	Yes
3. Were participants included in any comparisons similar?	Yes
4. Were the participants included in any comparisons receiving similar treatment/care, other than the exposure or intervention of interest?	Unclear
5. Were there multiple measurements of the outcome, both pre and post the intervention/exposure?	Yes
6. Were the outcomes of participants included in any comparisons measured in the same way?	Unclear
7. Were outcomes measured in a reliable way?	No
8. Was follow-up complete and if not, were differences between groups in terms of their follow-up adequately described and analysed?	Unclear
9. Was appropriate statistical analysis used?	Yes

Table S5. JBI Critical Appraisal Tool for Analytical Cross-Sectional Studies

Tool Items	Lewis, 2010
1. Were the criteria for inclusion in the sample clearly defined?	No
2. Were the study subjects and the setting described in detail?	Yes
3. Was the exposure measured in a valid and reliable way?	Unclear
4. Were objective, standard criteria used for measurement of the condition?	Unclear
5. Were confounding factors identified?	N/A
6. Were strategies to deal with confounding factors stated?	N/A
7. Were the outcomes measured in a valid and reliable way?	Yes
8. Was appropriate statistical analysis used?	Yes

Supplementary Materials

Table S6. Data synthesis: agreed themes sub-themes with definitions and coded items with quotes.

Theme & Definition	Sub-themes & Definitions	Codes	Quotes
**Accessibility to the Online Programme**<br><br>**Definition:** This theme explores carers' experiences and perceptions when accessing and using the programme. It includes both positive and negative aspects of the programme.	1. **Barriers and Challenges**<br><br>**Definition:** this sub-theme included carers negative experiences and perceived challenges, including technical difficulties, layout, design and format interactions during the programme and specific ways to access the programme.<br><br>2. **Facilitators and Opportunities**<br><br>**Definition**: this sub-theme pictured favourable aspects that enabled and enhanced carers' positive experiences, including flexibility and convenience of accessing the programme, clarity of instructions, user-friendliness.	1. Perceived challenges<br><br>2. Technical difficulties<br><br>3. Experience<br><br>4. Format and design	Lack of interaction with others was seen as a problem [ID2]<br>Carers reported having no time to adopt the app in daily life [ID7]<br>PwD found it difficult to learn how to use the app and use it independently [ID7]<br>Carers said PwD needed support to download apps, operating the overview of the app and navigating through categories [ID7]<br>The general format of the application was found unacceptable (technical specifications), with the smartphone option being the most difficult to use [ID3]<br>Carers experienced technical difficulties [ID2]<br>Carers found the directions clear [ID2]<br>Carers found the app easy to learn [ID7]<br>Carers found the DEM-DISC easy to learn [ID6]<br>Carers found the system user-friendly [ID6]<br>All carers found the portal was user friendly [ID4]<br>Carers thought DEM-DISC was easy to learn and user friendly [ID5]<br>Carers also found useful the possibility of using and accessing the programme at their own time and convenience [ID2]<br>PwD said they could easily remember how to use the app [ID7]<br>Carers reported increased interest in tablet devices [ID7]
**Online Programme Content**	1. **Engagement**<br><br>**Definition:** the extent to which carers actively interacted with and revisited various sections of the platform, indicating content relevance, interests, motivation and commitment.	1. Usefulness<br><br>2. Relevance and accomplishing<br><br>3. Sense of achievement	Most carers engaged with the content [ID1]<br>Carers appreciated the videoclips included in the programme [ID2]<br>The Forum was the most visited section of the programme [ID1]<br>Carers most frequently consulted area was related to practical problems and about the consequences of dementia [ID5]<br>Most carers found the platform useful and they found the support strategies module useful [ID4]<br>Carers rated the usefulness of DEM-DISC as moderate to neutral [ID6]

**Definition:** This theme encompasses carers' experiences with the content of the programme. It explores how carers received the content, met their expectations and contributed to their sense of achievement.	**2. Satisfaction**<br><br>**Definition:** carers' level of contentment and perceived usefulness of the platform, including positive and negative appraisal on the programme's structure and content.<br><br>**3. Comprehensiveness**<br><br>**Definition:** the extent to which the platform provided comprehensive and valuable information that met carers' needs, including the novelty of the content, suitability and amount of information provided.		The most useful module was practical difficulties in daily life and how to help, for the UK group [ID4]<br>The modules considered least useful were modules 1 (what is dementia?) and 3 (getting a diagnosis and why it is important) [ID4]<br>Carers enjoyed using an internet-based programme which held their interest and a third of the carers said the strategies identified were useful and liked by them [ID2]<br>Some carers were satisfied with DEM-DISC while neutral opinions were given to usefulness and satisfaction with DEM-DISC [ID5]<br>Both carers and PwD were satisfied with the app and agreed that the app was fun to use. However, carers were not sure that PwD would recommend the app to a friend [ID7] All carers found the pre training useful [ID7] but they thought the pre training should have been given in the presence of the PwD [ID7]<br>Carers were moderately satisfied with the programme [ID6]<br>General satisfaction of the carers with the application was unacceptable [ID3]<br>Carers had a qualified opinion of the programme. Some carers had negative opinion toward the programme and very few carers had a positive opinion [ID1]<br>Carers also mentioned that the pre training was not sufficient enough [ID7]<br>The most useful module for the Netherlands people was Living with dementia [ID4]<br>Specific elements of the portal were well received, such as the use of videos for the participants from the Netherlands and the interactive exercises and knowledge questions for the UK group [ID4]<br>Most carers agree that the programme contained the right amount of information [ID2]. However, others reported that the programme and content was repetitive and sometimes overwhelming [ID2]<br>The programme was considered useful, clear and comprehensive [ID1]<br>Half the carers found the pedagogical specifications of the application acceptable and the other half rated as unacceptable [ID3]
**Outcomes for the carer and the person with dementia**	1. **Skills, competence and carers' knowledge**<br><br>**Definition**: this sub-theme included those results for	1. Carer's development<br><br>2. Ideas and expertise	Carers gained new ideas and skills on how to care for the PwD [ID2]<br>Carers' Sense of Competence improved after using the programme [ID10]<br>Carers' sense of competence increased after the intervention [ID5]<br>Carers' sense of competence significantly increased after the intervention [ID9]<br>There was no improvement on the carers' sense of competence after

**Definition**: this theme explores and summarises the various outcomes for both carers and the people living with dementia as a result of completing the programme.	carers outcomes, including gaining skills, competence and knowledge.<br><br>**2. Activities of the person living with dementia**<br><br>**Definition**: this sub-theme describes the outcomes for the people with dementia's activities of daily living, including social participation, self-management, health response and autonomy.	3. Gains achieved by the person with dementia	completing the programme (6 months). After 12 months, the carers' sense of competence was higher in the experimental group [ID6]<br>Carers sense of competence did not improve after using the programme [ID8]<br>Carers sense of competence did not improve after the intervention [ID7]<br>There was no improvement on carer's competence after using the application [ID3]<br>Carers' sense of competence declined for those in the experimental group, after the intervention [ID4]<br>Knowledge increased but there was no improvement after 6 months follow-up [ID1]<br>Carers had a better understanding and knowledge of the disease [ID2]<br>Carers' knowledge about dementia did not improve after the intervention [ID4]<br>Carers knowledge about care and welfare did not improve after the intervention [ID5]<br>Higher educated PwD benefitted more from the App regarding social participation [ID8]<br>Social participation of the PwD did not improve after using the programme [ID10]<br>Social participation and engagement did not improve after using the programme [ID8]<br>PwD's social participation did not improve after the intervention [ID7] & [ID9]<br>PwD's engagement in pleasurable activities did not improve after the intervention [ID9]<br>PwD's health response did not improve after using the programme [ID10]<br>PwD's self-management abilities and experienced autonomy did not improve after using the programme [ID8]<br>PwD's perceived autonomy did not improve after the intervention (effect sizes may indicate possible positive influence of the programme on self-management and engagements in meaningful activities [ID7]<br>PwD's self-management abilities and experienced autonomy did not improve after the intervention [ID9]

ID1 Diapason (Cristancho-Lacroix, 2015). ID2 Internet-Based Savvy Caregiver (Lewis, 2010). ID3 UnderstAID (Núñez-Naveira, 2016). ID4 STAR-E Learning (Hattink, 2015). ID5 DEM-DISC (van der Roest, 2010). ID6 DEM-DISC (van Mierlo, 2015). ID7 FindMyApps (Kerkhof, 2022). ID8 FindMyApps (Beentjes, 2023). ID9 FindMyApps (Neal, 2023). ID10 FindMyApps (Neal, 2024).
